# Factors Affecting Perceived Work Environment, Wellbeing, and Coping Styles: A Comparison between Physicians and Nurses during the COVID-19 Pandemic

**DOI:** 10.3390/ijerph191711104

**Published:** 2022-09-05

**Authors:** Chiara Costa, Michele Teodoro, Annalisa De Vita, Federica Giambò, Carmela Mento, Maria Rosaria Anna Muscatello, Angela Alibrandi, Sebastiano Italia, Concettina Fenga

**Affiliations:** 1 Clinical and Experimental Medicine Department, University of Messina, 98125 Messina, Italy; 2 Department of Biomedical and Dental Sciences and Morphofunctional Imaging, Occupational Medicine Section, University of Messina, 98125 Messina, Italy; 3 Psychiatric Unit, Department of Biomedical and Dental Sciences and Morphofunctional Imaging, Clinical Psychology, University of Messina, 98125 Messina, Italy; 4 Psychiatric Unit, Department of Biomedical and Dental Sciences and Morphofunctional Imaging, University of Messina, 98125 Messina, Italy; 5 Department of Economics, University of Messina, 98125 Messina, Italy

**Keywords:** COVID-19, healthcare workers, work environment, wellbeing, coping strategies

## Abstract

The COVID-19 pandemic is a current emergency worldwide. All the consequent changes in sanitary systems have negatively affected the work–life balance. In particular, healthcare workers suffered from anxiety, stress, and depression, mostly nurses compared to physicians. To handle this situation, the adoption of different coping strategies has played a strategic role in psychophysical wellbeing. Our main goal is to the assess the perception of work environment and wellbeing (EQ-5D questionnaire), as well as to analyze possible differences in coping styles between physicians and nurses (brief COPE questionnaire). The arising differences were compared between the two groups, and associations with variables were assessed through a bivariate correlation analysis. This cross-sectional study was conducted from November to December 2020 through an online survey. A total of 172 respondents (117 physicians and 55 nurses), of which 102 were women and 70 were men, accepted to join the study. Our results showed that physicians referred a higher perception of wellbeing, and nurses reported an increased perception of work activity and efficiency, along with an unchanged economic status. The most frequently adopted coping strategies were Active and Planning (self-sufficient coping). Physicians showed a greater tendency to use avoidant coping strategies. More-experienced nurses and physicians were less prone to adopt socially supported coping strategies, emphasizing the need for novel organizational measures at the social dimension that favored sharing and interaction between peers. Future research should aim to further investigate the relationship between the perception of work environment and coping strategies in order to identify risk factors to be prevented by promoting adequate measures at an organizational level.

## 1. Introduction

The COVID-19 pandemic is an emergency that is still current in most countries, constituting an unprecedented challenge for populations and societies around the world [1].

National health systems did not have adequate levels of awareness to deal with the pandemic, and the absence of effective management models generated uncertainty in health organizations [2]. As a consequence, hospitals had to quickly adapt spaces and arrangements for the management of COVID-19 patients, as well as through the active recruitment of supplementary personnel [3]. Many healthcare workers (HCWs) were redeployed to areas outside their usual expertise, frequently working additional shifts and longer hours [4,5]. Despite these initial measures, HCWs had to work in a shortage of reliable personal protective equipment [6], a lack of specific COVID-19 protocols, and unsatisfactory instructions [7], with a higher risk of contracting COVID-19 than the general population [8]. Afterward, significant measures of public health for the prevention and control of COVID-19 were adopted and are continuously updated according to the current epidemiological situation.

All these changes have had a negative impact on the work environment, including personal working abilities, leadership, support between peers, resource adequacy, involvement in hospital plans, and quality of care [9]. In this scenario, the literature data suggest that the COVID-19 pandemic increased anxiety, stress, depression, and sleep disturbances, leading to a worsening of perceived wellbeing within the general population and, in particular, HCWs [10,11,12,13,14]. Wellbeing can be intended as a multidimensional concept, including physical, mental, and social determinants. It is well-known that, in the last few years, work demands have risen, reducing workers’ energies and resources to be spent in their social and private time [15,16].

In fact, physicians and nurses are the most-exposed to stressful situations [17] because the pandemic added further emotional and mental burdens related to the fear of contracting the virus and the worry of infecting family members, as well as a sense of helplessness in the management of patients [18].

Although the burdens of previous pandemics have been less impacting than the current one, HCWs have always experienced high rates of stress, anxiety, and mood alteration. Symptoms of post-traumatic stress have also been reported during the SARS outbreak. The external pressures may produce a dually harmful effect: at an individual level (with higher risk of burnout in HCWs) and at an organizational level. Altogether, these aspects may lead to adverse consequences for both patient management and healthcare system organization [19].

Differences in workload and professions are associated with stress [20,21] and, consequently, lower job satisfaction and performance, work accidents, absenteeism, and work-related stress [22,23]. Nurses have resulted as being the most-affected working category in hospital settings, showing higher levels of stress [4,23,24], difficulties in focusing at work [25], fear of uncontrollable virus spread, lower levels of trust in guidelines [24], depression, anxiety, and insomnia [26,27,28,29,30].

In this context, it is important to identify coping strategies that have been demonstrated to be helpful in controlling HCWs’ emotions, as well as in past pandemics [27]. The adoption of coping strategies, depending on external factors (such as culture and workplace context) [28] and subjective components (including emotions and mood status), plays a strategic role on psychological wellbeing [29]. On the other hand, discerning maladaptive reactions to stress might be useful for recognizing workers who may suffer from mental health problems.

The majority of the existing literature has focused on evaluating the perception of COVID-19 pandemic management among HCWs and the general population [30,31,32,33,34], whereas few investigations [35,36,37] have currently assessed the difference between physicians and nurses in the perception of wellbeing and stress-coping strategies, particularly focusing on whether the nursing profession might represent a risk factor itself or whether additional factors may also affect this vulnerability.

Under these premises, we hypothesize that the nursing profession might be associated with worse wellbeing perceptions [35]. Our main goal is to assess occurring differences between physician and nurse groups in perceptions of work environment and wellbeing, as well as in the adoption of group-specific coping strategies. Possible associations of wellbeing and coping strategies with social, demographic, and work-related features are also investigated.

## 2. Materials and Methods

### 2.1. Study Design and Population

This cross-sectional study was conducted from November to December 2020 through an online survey. Participants were physicians and nurses enrolled as hospital personnel working in a hospital based in the northeast of Sicily. An invitation link was sent to directors and coordinators requesting them to spread it to their staff, and data were collected through an online platform. The study population was divided into two groups: physicians (group P) and nurses (group N). A total of 172 respondents (n), 117 physicians and 55 nurses, accepted to join the study and completed the interview. A detailed sample description is summarized in Table 1. The number of women in group N was higher than in group P (67.3% and 55.6%, respectively), although this difference was not statistically relevant. Conversely, we found statistically significant differences in the other sociodemographic characteristics and work-related factors. The N population was older than the P population; in fact, about three-quarters of the subjects in group P (74.4%) were aged under 40 years, and the majority in group N (65.5%) was > 40 years. Over one-third (36.8%) of the respondents in group P were post-graduates, while 47 subjects (85.5%) in group N were graduates. Regarding marital status, in group P, single and married or cohabitant participants were similarly represented, while in group N, the majority had a partner (72.7%), and parenthood was more frequent in group N than in group P (72.7% and 29.1% had children, respectively). Considering work-related factors, one-third of nurses and only 10% of physicians were employed in COVID-19 wards; the majority of subjects in group P (54.7%) had no contact with COVID-19 patients, while most of the nurses (61.8%) had at least one contact per week with COVID-19 patients. Nurses had double the working seniority when compared with physicians (16 and 8 years, respectively).

This study was carried out in accordance with the ethical standards of the Helsinki Declaration. The study only needed a notification with a request for acknowledgement without formal approval by the local ethics committee. All the participants who accepted voluntary participation in the study provided informed consent. Participation was without compensation.

### 2.2. Procedures and Measures

The self-administered questionnaire was composed of two parts and took no more than fifteen minutes to be completed. The first part explored sociodemographic characteristics and work-related factors of the sample: gender, age, educational level, marital status, parenthood, employment in COVID-19 wards, number of contacts per week with COVID-19 patients, and work seniority. The second part consisted of the administration of two validated questionnaires to assess wellbeing perception and to evaluate coping strategies.

#### 2.2.1. Work Environment Perception

Participants were asked to indicate if they thought there was “reduction”, “no change” or “increment” in each category, including work activity (Q1), economic income (Q2), perception of work efficiency (Q3), and perception of work quality (Q4). Respondents’ feelings about their involvement in organizational changes or plans (Q5) were measured using a 4-point Likert-type scale, from 1 (never) to 4 (always). Subjects were also requested to evaluate (“Yes” or “No”) if the personal protective equipment (PPE) supply was adequate (Q6).

#### 2.2.2. Wellbeing Perception

To assess wellbeing perception, we used the European Quality of Life–5 Dimensions (EQ-5D) questionnaire. This instrument measures mobility, self-care, usual activities, pain or discomfort, and anxiety or depression through one inquiry for each dimension. Each question is scored from 1 to 3, in which 1 is “no difficulties”, 2 is “some difficulties”, and 3 is “many difficulties”. An algorithm permits the calculation of the EQ-5D index, in which 0 is death and 1 is perfect health. The EQ-5D is also composed of a Visual Analog Scale (VAS) measuring the subject’s perceived health status scored from 0 (the worst thinkable wellbeing) to 100 (the best thinkable wellbeing) [29]. In particular, the EQ-5D index score represents the health status, and the EQ-VAS gives information about individual health perception [38,39]. In the current investigation, Cronbach’s alpha for the EQ-5D Index was 0.59.

#### 2.2.3. Coping Strategies

The evaluation of different coping strategies was conducted through the administration of the Brief-COPE. This questionnaire assesses stress reaction in a recent period (“situational-actual” version). The tool includes 28 items scored from 1 to 4 according to a 4-point Likert scale and divided into 14 coping mechanisms, each consisting of two items. The 14 strategies are Active, Positive Reframing, Planning, Humor, Acceptance, Emotional Support, Instrumental Support, Venting, Religion, Self-Distraction, Substance Use, Denial, Disengagement, and Self-Blame [40]. Moreover, in order to allow a more accurate approach, we used a 3-factor structure, according to which the coping strategies were grouped in 3 dimensions: self-sufficient coping (Active, Positive Reframing, Planning, Humor, and Acceptance); socially supported coping (Emotional Support, Instrumental Support, Venting, and Religion,); and avoidant coping (Self-Distraction, Substance Use, Denial, Disengagement, and Self-Blame). Self-sufficient coping included emotion- and problem-focused strategies that were used to reduce the feelings of threat. Socially supported coping embraced mechanisms oriented toward the social environment. Finally, avoidant coping was the tendency to use behaviors toward rejection and disinterest during stressful situations [41]. In the present study, the reliability assessment for the different coping strategies showed the following Cronbach’s alpha values: Active, 0.70; Positive Reframing, 0.70; Planning, 0.74; Humor, 0.65; Acceptance, 0.54; Emotional Support, 0.81; Instrumental Support, 0.79; Venting, 0.58; Religion, 0.88; Self-Distraction, 0.50; Substance Use, 0.89; Denial, 0.55; Disengagement, 0.50; Self-Blame, 0.42; Self-sufficient Coping, 0.72; Socially Supported Coping, 0.71; and Avoidant Coping, 0.47.

### 2.3. Statistical Analysis

According to descriptive analyses, categorical variables were expressed as frequencies and proportions; continuous variables were expressed as means and standard deviations. To evaluate differences between physicians and nurses in categorical variables, we used a chi-squared test and Fisher’s exact test, as appropriate. After the application of the Kolmogorov–Smirnov test, all continuous variables followed a non-Gaussian distribution, although differences between groups were evaluated using the Mann–Whitney U test. The reliability of the questionnaires was evaluated through the computation of Cronbach’s alpha. Associations between variables were assessed through a bivariate correlation with Pearson’s correlation coefficient. *p*-values < 0.05 were considered statistically significant and reported in bold characters in the tables. Statistical analysis was performed using IBM SPSS Statistics 23 (IBM Corp, Armonk, NY, USA).

## 3. Results

### 3.1. Work Environment Perception

Regarding work environment perception, the answers from the interview are reported in Table 2. Under one-third of the physicians and about 40% of the nurses thought that work activity increased following the pandemic; 14.5% of the participants in group P and none in group N stated that their economic status increased; the majority of nurses (54.5%) and about one-third of the physicians (34.2%) had the perception of increased work efficiency. In questions from Q1 to Q3, the comparison between groups, conducted through a chi-squared test, underlined statistically significant differences. The majority of respondents in both groups reported an unchanged work quality and a low grade of involvement in organizational changes or plans. In addition, 84.6% of physicians and 78.2% of nurses stated that PPE supply was not adequate.

### 3.2. Wellbeing Perception

The assessment of wellbeing perception was carried out by the European Quality of Life–5 Dimensions (Index and VAS), whose scores are reported in Table 3. Despite the two groups showing high values of self-reported quality of life, group P showed better scores than group N both in the Index and VAS of the EQ-5D questionnaire, with statistically significant differences. Nurses showed worse scores than physicians in every component of the EQ-5D except anxiety or depression.

In group P, a bivariate correlation (Table 4) showed that the EQ-5D Index resulted in positive associations with graduation, less work seniority, higher work efficiency (Q3), and involvement in plans (Q5); the EQ-VAS was positively related to male gender, lower seniority, higher work efficiency (Q3), work quality (Q4), and involvement in plans (Q5). In group N, the same analysis (Table 4) showed that the EQ-5D Index resulted in positive associations with younger age, lower seniority, higher work efficiency (Q3), and perception of an adequate PPE supply (Q6); the EQ-VAS was positively related to younger age and lower seniority. Moreover, being a physician was positively associated with better scores in the EQ-5D Index (r = 0.237; *p*-value = 0.002) and EQ-VAS (r = 0.283; *p*-value = < 0.001).

### 3.3. Coping Strategies

Considering the mean scores of the Brief-COPE questionnaire (Table 5), the coping strategies with the highest scores were Active, Planning, Acceptance, and Positive Reframing (self-sufficient coping). Moreover, through the Mann–Whitney U test, we found statistically significant differences between physicians and nurses. In particular, physicians compared to nurses showed higher values in Self-Blame (*p*-value = 0.001) and Substance Use (*p*-value = 0.002) and lower values in Religion (*p*-value = 0.019) and Positive Reframing (*p*-value = 0.028).

In accordance with the Brief-COPE three-factor model, the results hereby obtained did not show any statistically relevant differences between the two groups.

In group P, a bivariate correlation (Table 6) showed that self-sufficient coping was positively associated with better perceived health status (EQ-VAS), lower levels of anxiety and depression, and increased work activity (Q1), work efficiency (Q3), work quality (Q4), and involvement in plans (Q5). Socially supported coping was related to lower wellbeing perception (EQ-5D Index), and higher levels of anxiety and depression. Avoidant coping was associated with being single, not having children, higher levels of anxiety and depression, increased perception of personal economic status (Q2), and lower work efficiency (Q3).

In group N, a bivariate correlation (Table 7) showed that self-sufficient coping was associated with a higher number of contacts per week with COVID-19 patients. Socially supported coping was correlated to female gender, graduation, lower seniority, and an increased perception of personal economic status (Q2). Avoidant coping did not show any statistically relevant associations.

## 4. Discussion

This study assessed the differences in the perceptions of work environment and wellbeing between physicians and nurses working in a hospital in southern Italy during the COVID-19 pandemic. Moreover, the adoption of different coping strategies was thoroughly analyzed. The potential association of wellbeing and coping strategies with social, demographic, and work-related features was also investigated.

Our results showed, overall, an unchanged perception of work environment and a positive perception of wellbeing. However, when clustering the sample based on profession, physicians referred a higher self-perceived wellbeing, while nurses reported an increased perception of work activity and efficiency, along with unchanged economic income. In addition, physicians showed a greater tendency to use avoidant coping strategies compared to nurses.

Regarding work environment features, approximately 40% of nurses reported an increased workload, while 30% of physicians perceived a reduction in work activity and quality during the first burst of the pandemic. This discrepancy between the two groups may be explained by nursing profession peculiarity, which involves providing care in COVID-19 units with direct contact with infected patients. Moreover, nurses had a high perception of work efficiency despite poor involvement in organizational plans. It could be hypothesized that the higher average seniority and, hence, the longer working experience of nurses present in this sample allowed them to handle these stressful situations using a balance of personal attitudes and beliefs, as well as professional skills [42,43]. Maybe this could be correlated to self-awareness of the important job performed by nurses during the pandemic, which could often be overwhelming [44].

In our population, the results of the validated questionnaires indicated that all the participants were aware of the benefits of their good health status and high quality of life, despite the comprehensible high level of anxiety and depression perceived. Currently, in agreement with other studies, physicians reported a higher perception of wellbeing compared to nurses [3,4,23,24]. In fact, being a physician was associated with better health status (r = 0.237; *p*-value = 0.002) and individual health perception (r = 0.283; *p*-value = < 0.001). A better wellbeing perception was positively associated with lower work seniority in both groups, similar to other research results [35,45]. One possible explanation might be found in a peculiarity of the healthcare profession, which is the presence of different stressors that could lead to the development of occupational burnout, hence reducing the overall quality of life. Moreover, increased workload and a reduction in work efficiency were worsening factors in wellbeing perception [46,47]. Altogether, these findings prove that perceived wellbeing is tightly correlated with the workplace as an essential component of individuals’ lives [48].

As mentioned above, the most-affected component of wellbeing was anxiety and depression, which showed broad associations with different coping strategies among physicians (Table 6). These findings are consistent with previous occupational health studies conducted among medical personnel [49,50]. It was demonstrated that the adoption of positive coping strategies could reduce anxiety and depression symptoms during the COVID-19 pandemic [51].

Seeking social support and avoidance are both primary coping mechanisms undertaken by individuals dealing with stress. Such coping strategies positively reduce the possible negative effects of stressful events by easing emotions [52,53]. In fact, in a study conducted on HCWs [27], over 70% of participants adopted the escape-avoidance mechanism, maybe due to the external pressure caused by the spread of COVID-19. The beneficial impacts of both social support and avoidance toward health and general wellbeing have been widely recognized. Specifically, previous studies have confirmed that social support directly provides wellbeing and promotes mental health, hence buffering the adverse effects of stressors. In addition, an attitude of avoidance as a self-protective mechanism can be actively adopted, for example, searching for diversions or company [17,54,55,56].

Overall, the most frequently adopted coping strategies were Active, Planning, Acceptance, and Positive Reframing in both groups. In particular, physicians showed a greater tendency to use avoidant coping strategies compared to nurses. It is possible that, due to professional characteristics, physicians are more prone to keep feelings to themselves, make an effort to forget, or avoid mental distress. These findings could be explained by a general feeling of overwhelming responsibility towards COVID-19 patients, which is not supported by adequate tools to manage it. These mechanisms have been demonstrated to be useful in the very first stages of stressful life events [57]. In the long run, with the persistence of a stressful situation, the effects of this self-defensive approach may become harmful, and it may be linked with the risk of developing depressive symptoms and burnout [58,59].

Sociodemographic characteristics, such as gender or parental status, were associated with coping mechanisms. In fact, female nurses relied more frequently on social support, especially when coming from close friends. Such support is important to modulate emotions, thus helping people in the positive management of stressful situations [60]. Physicians without children were more likely to use avoidant coping strategies, in contrast with another study in which being a parent was associated with the use of problem-focused coping [61]. Since the explanation for these results is not straightforward, other factors might mediate the currently observed association.

In particular, work-related factors and work environment perception played different roles in guiding the choice of coping strategies. Among nurses, an increased number of contacts per week with COVID-19 patients was positively associated to self-sufficient coping. Additionally, lower working seniority and higher economic income were positively associated with socially supported coping. These work-related factors, on an empirical basis, corresponded to the higher number of nurses exceptionally recruited to deal with the pandemic. These professionals, despite a lack of experience in managing stressors, as well as in problem solving and decision-making skills (which are vital when handling patient issues), adequately coped with the challenges related to the pandemic through seeking external support. Although the possibility of having social support was negatively affected by social isolation due to lockdown [62] and people reported difficulties in psychologically supporting each other [63], less-experienced nurses probably had the possibility of finding social support among peer colleagues.

On the other hand, the self-sufficient coping strategies used by doctors seemed to be more related to the perception of the work environment. Therefore, those who were more likely to adopt these strategies also had an increased perception of work efficiency and quality, as well as the feeling of being more involved in organizational changes or plans. It is well-known that coping strategies play an important role in the perception of work environment and, thus, in the prevention of work-related stress [64]. It is probable that the work environment has a greater influence on self-sufficient coping because physicians often may take independent decisions, so they might use socially supported coping strategies less often. Another study evidenced the importance of support groups where all workers could meet together and share their personal perceptions of work. In fact, a common problem observed in hospital environments is often poor communication between peers and superiors [65].

This study has also some limitations. Firstly, the cross-sectional design did not permit us to define the direction of causality. Secondly, the small size of the sample did not allow us to extend the results to the general HCW population. Finally, the data were collected retrospectively, and they had to be considered with prudence.

Despite these limitations, this survey highlighted different perceptions of work environment and personal wellbeing by physicians and nurses. It was also proved that coping strategies were implemented during the emergency period. The present study covered a gap in the current literature.

## 5. Conclusions

Our findings suggested that both individual and work-related factors were associated with life quality deterioration or with the adoption of non-functional coping strategies, regardless of job tasks.

These factors, if underestimated, may have repercussions on HCWs’ wellbeing, affecting mental health with consequent work impairment and job dissatisfaction. Lastly, this scenario could affect the quality of HCWs’ care, as well as increase the occurrence of errors and potential injuries.

More-experienced nurses and physicians were less prone to adopt socially supported coping strategies, highlighting a need for the implementation of organizational measures in the social dimension in order to favor both sharing and interaction between peers.

Future research should aim to further investigate the relationship between the perception of work environment and chosen coping strategies in order to identify risk factors that might be prevented by promoting adequate measures at an organizational level. 

## Figures and Tables

**Table 1:** Description of study population: sociodemographic characteristics and work-related factors (n = 172).

		Total	Group P	Group N	
		n (%)	n (%)	n (%)	*p*-Value
Sociodemographic Factors					
Total		172 (100)	117 (68.0)	55 (32.0)	
Gender					
	Male	70 (40.7)	52 (44.4)	18 (32.7)	0.145
	Female	102 (59.3)	65 (55.6)	37 (67.3)	
Age					
	<40 y	106 (61.6)	87 (74.4)	19 (34.5)	**<0.001**
	>40 y	66 (38.4)	30 (25.6)	36 (65.5)	
Education					
	Post-graduation	51 (29.7)	43 (36.8)	8 (14.5)	**0.003**
Marital status					
	Single	75 (43.6)	60 (51.3)	15 (27.3)	**0.003**
	Married or cohabitant	97 (56.4)	57 (48.7)	40 (72.7)	
Parenthood					
	No	98 (57.0)	83 (70.9)	15 (27.3)	**<0.001**
	Yes	74 (43.0)	34 (29.1)	40 (72.7)	
Work-Related Factors					
COVID-19 ward					
	No	142 (82.6)	106 (90.6)	36 (65.5)	**<0.001**
	Yes	30 (17.4)	11 (9.4)	19 (34.5)	
Number of contacts per week with COVID-19 patients					
	None	85 (49.4)	64 (54.7)	21 (38.2)	**0.033**
	One	25 (14.5)	18 (15.4)	7 (12.7)	
	Five	41 (23.8)	26 (22.2)	15 (27.3)	
	Exclusive	21 (12.2)	9 (7.7)	12 (21.8)	
Seniority (years)					
	Mean ± SD	10.67 ± 10.23	7.97 ± 8.86	16.44 ± 10.44	**0.012**

Group P includes physicians; group N includes nurses. Percentages are compared through chi-squared tests; means are compared through Mann–Whitney U tests. Statistically significant *p*-values are reported in bold characters.

**Table 2:** Description of the interview results regarding work environment during COVID-19 pandemic (n = 172).

	Total n (%)	Group P n (%)	Group N n (%)	*p*-Value
Q1. Your work activity is…				
Reduced	53 (30.8)	46 (39.3)	7 (12.7)	**0.002**
Unchanged	66 (38.4)	39 (33.3)	27 (49.1)	
Increased	53 (30.8)	32 (27.4)	21 (38.2)	
Q2. Your economic income is…				
Reduced	17 (9.9)	9 (7.7)	8 (14.5)	**0.007**
Unchanged	138 (80.2)	91 (77.8)	47 (85.5)	
Increased	17 (9.9)	17 (14.5)	0 (0.0)	
Q3. Your perception of work efficiency is…				
Reduced	46 (26.7)	35 (29.9)	11 (20.0)	**0.040**
Unchanged	56 (32.6)	42 (35.9)	14 (25.5)	
Increased	70 (40.7)	40 (34.2)	30 (54.5)	
Q4. Your perception of work quality is…				
Reduced	70 (40.7)	54 (46.2)	16 (29.1)	0.076
Unchanged	75 (43.6)	48 (41.0)	27 (49.1)	
Increased	27 (15.7)	15 (12.8)	12 (21.8)	
Q5. How much do you feel involved in organizational changes or plans?				
≤2	109 (63.4)	74 (63.2)	35 (63.6)	0.961
>2	63 (36.6)	43 (36.8)	20 (36.4)	
Mean ± SD	2.2 ± 1.0	2.2 ± 0.9	2.2 ± 1.1	0.873
Q6. Do you think the PPE supply is adequate?				
Yes	30 (17.4)	18 (15.4)	12 (21.8)	0.300
No	142 (82.6)	99 (84.6)	43 (78.2)	

Group P includes physicians; group N includes nurses. Percentages are compared through Chi-squared test; means are compared through Mann-Whitney U test. Statistically significant *p*-values are reported in bold characters.

**Table 3:** Mean scores of validated questionnaires assessing health-related factors and perceived quality of life during the first wave of the COVID-19 pandemic (n = 172).

		Total	Group P	Group N	
		Mean ± SD	Mean ± SD	Mean ± SD	*p*-Value
EQ-5D Index		0.825 ± 0.231	0.862 ± 0.192	0.745 ± 0.285	**0.009**
EQ-VAS		78.20 ± 18.15	81.71 ± 13.71	70.73 ± 13.58	**0.005**
EQ-5D components					
	Mobility	1.10 ± 0.31	1.06 ± 0.24	1.20 ± 0.40	**0.005**
	Self-care	1.02 ± 0.13	1.00 ± 0.00	1.05 ± 0.23	**0.011**
	Usual activities	1.16 ± 0.37	1.10 ± 0.31	1.27 ± 0.45	**0.004**
	Pain or discomfort	1.27 ± 0.47	1.17 ± 0.38	1.49 ± 0.57	**<0.001**
	Anxiety or depression	1.46 ± 0.59	1.47 ± 0.60	1.44 ± 0.57	0.769

Group P includes physicians; group N includes nurses. Means are compared through Mann–Whitney U test. Statistically significant *p*-values are reported in bold characters.

**Table 4:** Correlation matrix between European Quality of Life questionnaire and sociodemographic characteristics, work-related factors, and work environment perception in group P and group N.

	Group P		Group N	
	EQ-5DIndex	EQ-VAS	EQ-5DIndex	EQ-VAS
Gender	r = 0.153	r = 0.203 *	r = 0.139	r = 0.219
Age	r = −0.181	r = −0.131	r = −0.291 *	r = −0.280 *
Education	r = −0.219 *	r = −0.050	r = 0.233	r = 0.186
Marital status	r = −0.047	r = −0.122	r = −0.055	r = 0.150
Parenthood	r = −0.098	r = −0.080	r = −0.257	r = −0.156
COVID-19 ward	r = 0.035	r = 0.035	r = −0.084	r = −0.145
N° contacts per week	r = −0.037	r = 0.137	r = −0.160	r = −0.058
Work seniority	r = −0.271 **	r = −0.212 *	r = −0.417 **	r = −0.432 **
Q1 (work activity)	r = −0.166	r = 0.011	r = −0.057	r = −0.234
Q2 (economical status)	r = 0.112	r = 0.089	r = 0.047	r = 0.145
Q3 (work efficiency)	r = 0.234 **	r = 0.228 **	r = 0.322 *	r = 0.080
Q4 (work quality)	r = 0.120	r = 0.237 **	r = 0.234	r = 0.096
Q5 (involvement in plans)	r = 0.215 *	r = 0.211 *	r = 0.209	r = 0.134
Q6 (PPE)	r = −0.076	r = 0.007	r = 0.277 *	r = 0.191

Bivariate correlation analysis with Pearson’s coefficient calculation was performed. (* for *p* < 0.05 and ** for *p* < 0.01). Statistically significant *p*-values are reported in bold characters.

**Table 5:** Mean scores of Brief-COPE during the first wave of the COVID-19 pandemic (n = 172).

		Total	Group P	Group N	
		Mean ± SD	Mean ± SD	Mean ± SD	*p*-Value
Brief-COPE					
	Active	6.53 ± 1.52	6.59 ± 1.42	6.42 ± 1.71	0.774
	Positive Reframing	5.47 ± 1.65	5.28 ± 1.68	5.85 ± 1.55	**0.028**
	Planning	6.55 ± 1.46	6.60 ± 1.42	6.45 ± 1.55	0.651
	Humor	3.99 ± 1.58	4.06 ± 1.65	3.84 ± 1.41	0.560
	Acceptance	6.13 ± 1.48	6.10 ± 1.49	6.20 ± 1.46	0.722
	Emotional Support	4.49 ± 1.73	4.64 ± 1.77	4.16 ± 1.62	0.135
	Instrumental Support	4.84 ± 1.84	4.96 ± 1.84	4.60 ± 1.83	0.286
	Venting	4.32 ± 1.59	4.44 ± 1.59	4.07 ± 1.59	0.147
	Religion	3.95 ± 1.91	3.70 ± 1.78	4.49 ± 2.08	**0.019**
	Self-Distraction	5.30 ± 1.56	5.41 ± 1.49	5.07 ± 1.70	0.233
	Substance Use	2.35 ± 1.01	2.50 ± 1.17	2.05 ± 0.41	**0.002**
	Denial	3.06 ± 1.38	2.89 ± 1.34	3.42 ± 1.41	**0.006**
	Disengagement	2.87 ± 1.32	2.93 ± 1.39	2.75 ± 1.17	0.496
	Self-Blame	5.39 ± 1.56	5.68 ± 1.52	4.78 ± 1.47	**0.001**
Brief-COPE three-factor model					
	Self-sufficient	28.67 ± 5.30	28.63 ± 5.22	28.76 ± 5.51	0.765
	Socially-supported	17.60 ± 5.17	17.74 ± 5.17	17.33 ± 5.19	0.725
	Avoidant	18.98 ± 3.93	19.40 ± 4.28	18.07 ± 2.85	0.076

Group P includes physicians; group N includes nurses. Means are compared through Mann–Whitney U test. Statistically significant *p*-values are reported in bold characters.

**Table 6:** Correlation matrix between Brief-COPE and sociodemographic characteristics, work-related factors, European Quality of Life, and work environment perception in group P.

	Self-Sufficient Coping	Socially Supported Coping	Avoidant Coping
Gender	r = 0.007	r = −0.054	r = −0.088
	*p* = 0.940	*p* = 0.562	*p* = 0.344
Age	r = 0.008	r = 0.030	r = −0.101
	*p* = 0.935	*p* = 0.746	*p* = 0.278
Education	r = −0.025	r = 0.012	r = −0.059
	*p* = 0.793	*p* = 0.901	*p* = 0.525
Marital status	r = 0.076	r = −0.086	**r = −0.192**
	*p* = 0.418	*p* = 0.357	***p* = 0.038**
Parenthood	r = −0.071	r = −0.110	**r = −0.294**
	*p* = 0.449	*p* = 0.239	***p* = 0.001**
COVID-19 ward	r = 0.023	r = 0.159	r = 0.045
	*p* = 0.807	*p* = 0.087	*p* = 0.629
N° contacts per week	r = 0.012	r = −0.065	r = −0.088
	*p* = 0.895	*p* = 0.484	*p* = 0.345
Work seniority	r = −0.011	r = 0.019	r = −0.070
	*p* = 0.910	*p* = 0.842	*p* = 0.451
EQ-5D Index	r = 0.180	**r = −0.200**	r = −0.164
	*p* = 0.052	***p* = 0.030**	*p* = 0.076
EQ-VAS	**r = 0.213**	r = 0.025	r = −0.063
	***p* = 0.021**	*p* = 0.787	*p* = 0.499
Mobility	r = −0.038	r = 0.069	r = −0.032
	*p* = 0.687	*p* = 0.460	*p* = 0.730
Self-care	r = 0.054	r = 0.126	r = 0.024
	*p* = 0.852	*p* = 0.951	*p* = 0.746
Usual Activities	r = 0.018	r = 0.023	r = 0.067
	*p* = 0.843	*p* = 0.807	*p* = 0.471
Pain or discomfort	r = 0.045	r = 0.094	r = −0.043
	*p* = 0.628	*p* = 0.314	*p* = 0.647
Anxiety or depression	**r = −0.299**	**r = 0.239**	**r = 0.284**
	***p* = 0.001**	***p* = 0.009**	***p* = 0.002**
Q1 (work activity)	**r = 0.203**	r = 0.033	r = −0.118
	***p* = 0.028**	*p* = 0.720	*p* = 0.207
Q2 (economical status)	r = 0.007	r = 0.104	**r = 0.214**
	*p* = 0.942	*p* = 0.266	***p* = 0.021**
Q3 (work efficiency)	**r = 0.210**	r = −0.053	**r = −0.273**
	***p* = 0.023**	*p* = 0.568	***p* = 0.003**
Q4 (work quality)	**r = 0.182**	r = −0.061	r = −0.160
	***p* = 0.049**	*p* = 0.515	*p* = 0.084
Q5 (involvement in plans)	**r = 0.279**	r = 0.166	r = −0.045
	***p* = 0.002**	*p* = 0.074	*p* = 0.631
Q6 (PPE)	r = −0.043	r = −0.098	r = −0.107
	*p* = 0.647	*p* = 0.295	*p* = 0.252

Bivariate correlation analysis with Pearson’s coefficient calculation was performed. Statistically significant *p*-values are reported in bold characters.

**Table 7:** Correlation matrix between Brief-COPE and sociodemographic characteristics, work-related factors, European Quality of Life, and work environment perception in group N.

	Self-Sufficient Coping	Socially Supported Coping	Avoidant Coping
Gender	r = 0.098	**r = −0.353**	r = −0.059
	*p* = 0.478	***p*** **= 0.008**	*p* = 0.668
Age	r = 0.004	r = −0.207	r = 0.046
	*p* = 0.979	*p* = 0.130	*p* = 0.740
Education	r = 0.188	**r = 0.345**	r = 0.154
	*p* = 0.169	***p*** **= 0.010**	*p* = 0.263
Marital status	r = 0.175	r = 0.166	r = 0.117
	*p* = 0.200	*p* = 0.226	*p* = 0.396
Parenthood	r = 0.198	r = 0.039	r = −0.013
	*p* = 0.148	*p* = 0.778	*p* = 0.924
COVID-19 ward	r = 0.179	r = 0.192	r = -0.032
	*p* = 0.192	*p* = 0.161	*p* = 0.815
N° contacts per week	**r = 0.339**	r = 0.125	r = −0.012
	***p*** **= 0.011**	*p* = 0.363	*p* = 0.928
Work seniority	r = −0.099	**r = −0.316**	r = 0.071
	*p* = 0.472	***p*** **= 0.019**	*p* = 0.607
EQ-5D Index	r = 0.052	r = 0.052	r = −0.101
	*p* = 0.707	*p* = 0.705	*p* = 0.463
EQ-VAS	r = 0.121	r = 0.007	r = −0.056
	*p* = 0.378	*p* = 0.959	*p* = 0.685
Mobility	r = −0.053	r = −0.253	r = 0.019
	*p* = 0.699	*p* = 0.063	*p* = 0.889
Self-care	r = −0.063	r = −0.124	r = 0.164
	*p* = 0.648	*p* = 0.366	*p* = 0.232
Usual Activities	r = −0.026	r = −0.031	r = 0.071
	*p* = 0.851	*p* = 0.822	*p* = 0.607
Pain or discomfort	r = −0.027	r = −0.030	r = 0.046
	*p* = 0.844	*p* = 0.827	*p* = 0.740
Anxiety or depression	r = −0.161	r = 0.032	r = 0.060
	*p* = 0.239	*p* = 0.815	*p* = 0.664
Q1 (work activity)	r = 0.027	r = −0.173	r = 0.087
	*p* = 0.847	*p* = 0.207	*p* = 0.529
Q2 (economical status)	r = 0.218	**r = 0.297**	r = 0.138
	*p* = 0.109	***p*** **= 0.028**	*p* = 0.314
Q3 (work efficiency)	r = 0.095	r = 0.155	r = −0.068
	*p* = 0.492	*p* = 0.257	*p* = 0.621
Q4 (work quality)	r = 0.014	r = 0.216	r = −0.043
	*p* = 0.917	*p* = 0.114	*p* = 0.757
Q5 (involvement in plans)	r = 0.022	r = 0.237	r = 0.126
	*p* = 0.875	*p* = 0.082	*p* = 0.360
Q6 (PPE)	r = −0.066	r = −0.111	r = 0.095
	*p* = 0.633	*p* = 0.421	*p* = 0.488

Bivariate correlation analysis with Pearson’s coefficient calculation was performed. Statistically significant *p*-values are reported in bold characters.

## Data Availability

The datasets generated and analyzed during the current study are available from the corresponding author on reasonable request.
